# Watch Heart in Lobar Pneumonia

**Published:** 1918-10

**Authors:** 


### Watch Heart in Lobar Pneumonia.

Deaths of pneumonia patients are due either to heart failure or to septicemia, seldom, if ever, to insufficient aeration of the blood in the lungs says Dr. E. P. Hershey of Denver in his prize answer to the New York Medical Journal's question, "How do you treat lobar pneumonia?"

Dr. Hershey says the heart must be watched and stimulated with digitalis. If it can be obtained the appropriate vaccine is to be used. In spite of prejudice, he recommends the ice bag. If the patient be alcoholic he must have whisky or brandies; if not, all liquor must be cut out. A generous diet, but without meat, is necessary. Open air treatment is conquering ancient prejudice. Sudden rise of temperature on the third, fifth or seventh day is no cause for alarm, and depressing medicines at this time may mean death.

---

Dr. Carnes, city health officer of Dallas, advises that all dogs and cats be interned during the prevalence of Spanish influenza, as they are carriers of the germs. Dr. Carnes used the wrong word; he should have advised that all dogs and cats be interred. They are a nuisance, an expense and a menace to health. The money that is wasted in taking care of useless dogs and cats in this country would keep the babies in Belgium in comfort.

---

Dr. J. Breckenridge Bayne of Washington, D. C., after having been held a prisoner for a time by the Germans, has been decorated by King Ferdinand of Roumania for his effective work in fighting a typhus epidemic in that country.

